# Krill vs salps: dominance shift from krill to salps is associated with higher dissolved N:P ratios

**DOI:** 10.1038/s41598-020-62829-8

**Published:** 2020-04-03

**Authors:** Christoph Plum, Helmut Hillebrand, Stefanie Moorthi

**Affiliations:** 1 University of Oldenburg, Institute for Chemistry and Biology of the Marine Environment (ICBM), Wilhelmshaven, Germany; 2 Helmholtz Institute for Functional Marine Biodiversity (HIFMB) at the University of Oldenburg, Oldenburg, Germany; 3 Alfred Wegener Institute, Helmholtz-Centre for Polar and Marine Research (AWI), Bremerhaven, Germany

**Keywords:** Element cycles, Element cycles, Marine biology

## Abstract

Pronounced atmospheric and oceanic warming along the West Antarctic Peninsula (WAP) has resulted in abundance shifts in populations of Antarctic krill and *Salpa thompsoni* determined by changes in the timing of sea-ice advance, the duration of sea-ice cover and food availability. Krill and salps represent the most important macrozooplankton grazers at the WAP, but differ profoundly in their feeding biology, population dynamics and stoichiometry of excretion products with potential consequences for the relative availability of dissolved nitrogen and phosphorus. Alternation of the dissolved nutrient pool due to shifts in krill and salp densities have been hypothesized but never explicitly tested by using observational data. We therefore used the Palmer LTER dataset in order to investigate whether the dominance of either grazer is related with the observed dissolved nitrogen:phosphorus (N:P) ratios at the WAP. Across the whole sampling grid, the dominance of salps over krill was significantly correlated to higher concentrations of both N and P as well as a higher N:P ratios. Using actual long-term data, our study shows for the first time that changes in key grazer dominance may have consequences for the dynamics of dissolved nitrogen and phosphorus at the WAP.

## Introduction

The ocean around the West Antarctic Peninsula (WAP) is a highly productive system with summer peak phytoplankton blooms promoting large populations of macrozooplankton such as the Antarctic krill *Euphausia superba*, the salp *Salpa thompsoni* (the predominant pelagic tunicate in this region) and many marine mammals^1–3^. The dynamics of this unique ecosystem are commonly driven by the seasonal extent and retreat of sea ice and its interannual variability^4^. However, the Antarctic Peninsula has experienced major alterations in climate conditions during the last decades^4^. The rapid warming of winter air temperature and sea surface temperature resulted in declining perennial and seasonal sea-ice extent and duration^5–9^. Since this warming effect is more pronounced in the northern half of the peninsula, the latitudinal climate gradient with warmer, humid conditions in the north but a cold-dry polar-type continental climate in the south has become steeper^7–9^. These alterations have caused considerable changes in the phytoplankton community^2,10^. While algal biomass dramatically decreased in the northern region of the WAP involving changes in community composition from large diatoms to smaller flagellate species, phytoplankton biomass increased in the southern part due to the expanding open water areas associated with the decline in sea-ice cover^2,10–12^.

The observed changes in the abiotic environment and phytoplankton community structure have a strong potential to influence reproduction, recruitment and distribution of key grazers such as *Euphausia superba* (krill hereafter) and the salp *Salpa thompsoni* (salps) due to a strong bottom-up control of the system^9^. The temporal and spatial fluctuations in the abundance of krill and salps are commonly assigned to water temperatur, the timing of sea-ice advance, duration of sea-ice cover and food availability^13,14^. Krill abundance, in particular, is tightly coupled to the sea-ice formation, predominantly in areas of krill recruitment, as well as higher concentrations of chlorophyll^14,15^. In contrast, *S. thompsoni* is considered as a typical oceanic species, favoring warmer and ice-free open waters with lower food concentrations^13,16,17^.

During the last decades, several publications have reported a shift in the distribution and abundance of krill and salps in the Southern Ocean^3,12,18–23^. Previous studies focusing on large-scale spatial and temporal dynamics of the krill population by using the KRILLBASE dataset have reported a considerable decrease in krill density and simultaneous southward shift of the population within the SW Atlantic sector of the SO^18^. Although another study suggested that the decline is less severe than predicted^24^, a recently published model confirmed the previously described trend in decreasing krill density especially north of the WAP^21^. In addition, the shrinking krill population is further contracting southward and closer to the shelf^21^, eventually being replaced by salps which intrude the warmer, ice-free water bodies^16,22,23^.

Changes in the frequency of salp and krill occurrence as well as their abundance have been also observed along the western shores of the Antarctic Peninsula^20,25^. However, the long-term trend in krill and salp abundance is less clear. Considering solely the data available from the Palmer LTER grid, a monitoring programm covering 30 years of observations stretching along the WAP, no overall long-term directional trend in krill and salp density has been observed so far^3,25^. Here, anomalies in krill and salp abundance were less pronounced or even opposite to those observed north of the peninsula^25^. Spatial and temporal overlap of krill and salps has became more common in recent years, but neither salp peak densities nor the frequency of peak years with high salp abundance increased along the WAP^17,22,25,26^. Salp abundance alternated between negative and positive anomalies with no long-term changes in the northern part of the Palmer grid. However, as with krill, highest salp densities shifted southward with an increasing trend in abundance with more and larger positive anomalies in the second half of the Palmer LTER time-series^3,25^.

Potential long-term shifts in krill and salp populations may have a significant impact on phytoplankton community structure, food web dynamics and the biogeochemistry of the WAP pelagic ecosystem. Antarctic krill in particular plays a major role in the system, as it represents a direct link between primary producers and higher trophic levels^27,28^. While the importance of salps as a food source for higher trophic levels is controversial^29^, their rapid formation of high densities and their high grazing efficiencies can have substantial impacts on primary production^30^. As important macrograzers, krill and salps not only directly influence phytoplankton communities via grazing, but also indirectly through the remineralisation and resupply of macronutrients such as nitrogen and phosphorus that are pivotal for phytoplankton to thrive^31–33^. Several studies have highlighted the importance of zooplankton nutrient recycling in the marine environment of the SO^31,34–37^. While different abundant zooplankton groups such as copepods, amphipods or pteropods as well as microzooplankton contribute to the recycling of nutrients and trophic interactions around the WAP, Antarctic krill and *S. thompsoni* are generally recognized as key species for the remineralization of inorganic nutrients and the transfer of organic matter^32,36,38–43^.

Two mechanisms of recycling are commonly proposed for meso- and macrozooplankton – egestion and subsequent degradation of particulate organic matter in form of fecal pellets and the excretion of inorganic nutrients such as nitrogen and phosphorus. However, Antarctic krill and salps reveal profound differences in their body stoichiometry, fecal pellet production and excretion rates. Although the previously reported excretion rates of *E. superba* and *S. thompsoni* show a high variability^34,44,45^, the average carbon specific metabolic rates of N and P as well as the N:P excretion ratios of salps can be higher than those observed for krill and copepods^41,46^. Such interspecific differences in the stoichiometry of consumer excretion products can substantially modify the nutrient supply to primary producers by changing the relative availability of essential macronutrients, which in turn can result in strong indirect effects on primary production and community composition^47–49^. Accordingly, a previous model based on experimentally obtained data of individual nutrient excretion rates and respiration predicted that a shift from krill to salps may significantly change the C, N, and P cycles in the Southern Ocean^41^. Hence, one might expect that an increasing occurance of salps at the WAP may lead to a shift towards higher N:P ratios in the dissolved nutrient pool. Subsequently, phytoplankton community structure as well as trophic interactions could be modified due to elemental imbalances between planktonic consumers and their food with consequences for whole food web dynamics.

However, data on krill and salp stoichiometry as well as on dissolved N and P in the SO are still scarce, limiting valid information on potential long-term effects of shifts in krill and salp populations on the dynamics of dissolved nutrients. While potential top-down effects of the observed changes in krill and salp abundance on nutrient dynamics at the WAP have been hypothesized and discussed in previous studies^37,41,50^, the relationship between krill and salp density and the N and P dynamics has never been explicitly investigated by using environmental long-term datasets. In order to evaluate previous assumptions and to predict potential consequences of a long-term shift from krill to salps on nutrient dynamics along the WAP we analyzed 23 years of the Palmer Long-Term Environmental Research (Palmer LTER) time-series dataset with regard to the relationship between salp and krill abundance and the dissolved N and P concentrations as well as dissolved N:P ratios. Based on the assumption that salps and krill differ in their excretion stoichiometry (i.e. salps excrete more N than krill in relation to P), we expect a significant positive relationship between higher salp densities and the availability of dissolved N, P, and the N:P ratio.

## Material and Methods

**Figure 1:**
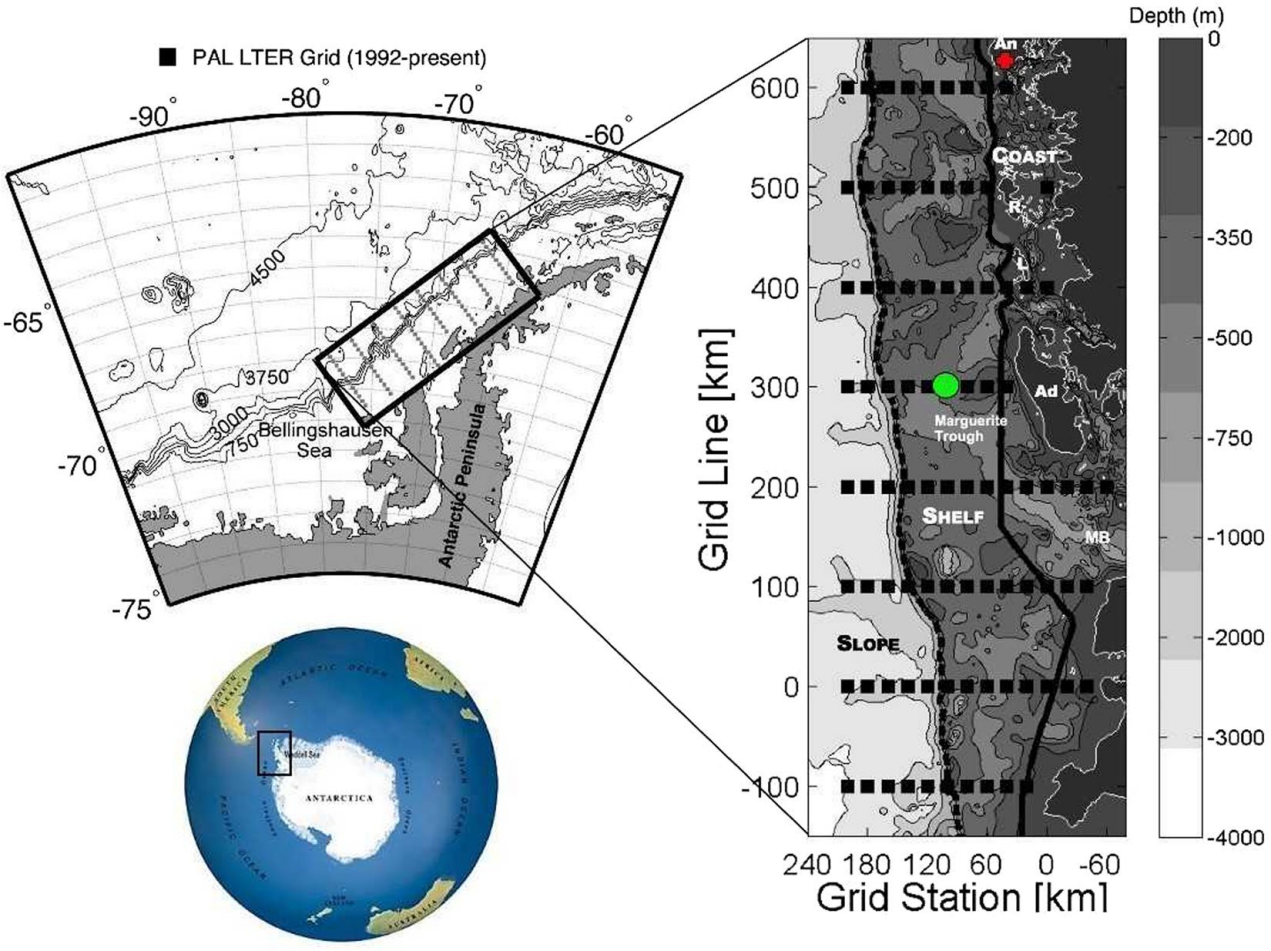
Palmer LTER study area location and bathymetry. Stations are located on a grid system (shown within the rectangular box) off the West Antarctic Peninsula. Bathymetry is indicated by grey shading, with depths noted in the greyscale color bar to the right. The grid encompasses continental slope, shelf and coastal regions as separated (and labeled) by dashed and solid lines running roughly parallel to the coast. Position of the physical oceanographic mooring is shown by the green circle. The red dot represents the location of U.S. Palmer Station. An = Anvers Island (home of Palmer Station), R = Renaud Island, L = Lavosier Island, Ad = Adelaide Island (home of U.K. Rothera Station), MB = Marguerite Bay. Figure adapted from Palmer LTER network. Figure courtesy D. Martinson and R. Iannuzzi, Palmer Antarctica LTER.

To assess the potential effect of krill and salps on the availability of dissolved N, P and the N:P ratio, we used the Palmer LTER long-term dataset (https://pal.lternet.edu/data). The Palmer LTER study area is situated at the west-coast of the Antarctic Peninsula and reaches from Anvers Island in the north to approx 700 km south near Charcot Island and from coastal to slope waters approx. 200 km offshore (Fig. 1). The Palmer LTER grid was sampled each year during annual research cruises in austral summer (January-February) aboard the MV Polar Duke and ARSV Laurence M Gould. Consequently, the data we used refer solely to the summer peak season.

From the Palmer cruise data time-series, we extracted the historical and current zooplankton densities as well as the dissolved inorganic nutrient data including dissolved N and P collected between 1991 and 2016. Detailed information on zooplankton collection and nutrient sampling processes have been described in previous studies and reports^51–53^.

For our analysis, we excluded samples with no or zero concentrations of dissolved nutrients (i.e., we only included samples with concentrations >0). Some samples showed very high concentrations, therefore we deleted outliers defined as nutrient concentrations outside of 2.5*standard deviation.

The majority of dissolved nutrient data was obtained from depths that matched the zooplankton hauls from 120 m. In most cases, only two nutrient sampling depths per station were deeper than the permanent pycnocline (150–200 m). However, we decided to include those observations in our analysis for the following reasons. Nutrient concentrations below 100 m revealed very low variability. Consequently, excluding these values from the analysis had no significant effect on the outcome. In addition, krill and in particular salps show a pronounced vertical migration and have been observed at depth deeper than 150 m^30,39,54^. As a consequence, zooplankton migration may contribute to nutrient fluxes across the pycnocline to depth deeper than 120 m.

The nutrient data of the Palmer LTER were measured multiple times at the same station in the same year, but this was not consistent across all variables and time periods. Therefore, we used mean values and combined the mean zooplankton data with the mean nutrient data based on station number, grid line number and year in order to be able to compare the density data with the nutrient variables. As a consequence, the dataset was reduced down to 823 observations across the whole Palmer grid between 1993 and 2016. In the reduced dataset, density values for the years 1994, 1997, 1998, 2009 and 2010 were partly excluded by merging the zooplankton data with the environmental data due to missing N and P measurements. An additional figure of the whole dataset between 1993 and 2016 is included in the appendix (Appendix Fig. 1).

In accordance with previous zooplankon analyses using the Palmer dataset^8^, we either included the entire grid for our analysis, or divided the grid into latitudinal sub-regions based on hydrographic and sea-ice conditions. We partly adapted the methods from Steinberg *et al*.^8^ but divided the grid into North (sampling lines 400–600) and South (sampling lines −100–300). We also considered the bathymetric gradient from the coast to the oceanic regions by dividing the grid into coastal stations (<500 m), shelf stations (500–1000 m) and the slope region (>1000 m)^8^.

In addition, krill and salp abundance data were corrected for differences between day and night catches in previous studies^3,20,55^. Based on this, we assessed the potential effect of day vs. night sampling on the outcome of our analyses. Similar to the previous investigations, we determined sun elevation at the time and location of each tow, with night defined as a sun elevation < −0.833° ^3,20,55^. We then tested for differences between night and day densities by using an analysis of variance (ANOVA) on krill and salp abundance data. As we determined no significant differences in our dataset between day and night catches, we did not correct the densities for further analyses. Similarly, Steinberg *et al*.^3^ stated, that the use of corrected or uncorrected data had no significant effect on their results. In addition, another previous study demonstrated that the effect of corrected daytime krill densities was minor for December and January^55^. Therefore, we decided to test our hypothesis on uncorrected density data.

### Statistical analysis

In order to determine the relationship between krill vs. salps and the concentrations of dissolved N and P as well as the corresponding molar N:P ratio we performed a linear mixed effects model (LMM) using the lmer function in the R package *lme4*^56^. As random effects, we used year and the Palmer LTER stations nested in year to account for the interannual and spatial variation in the data. This allowed for different intercepts by the temporal and spatial location of the sample. As fixed effects, we entered the salp and krill abundance as interacting terms into the model. Whereas this analysis tested for the effects of salp and krill density (Model 1), we ran an additional model with only the salp:krill ratio as fixed effect (Model 2). We conducted all analyses on log transformed data to meet the assumptions of normality. We assumed a Gaussian distribution and checked the normal distribution of model residuals to confirm goodness of fit. We additionally checked the residual plots to ensure homoscedasticity. Based on the F-ratios obtained for the fixed factors in the LMM, we obtained significance levels using the critical F for the appropriate degrees of freedom. We initially included other abundant zooplankton taxa such as the gastropod *Limacina* and copepods in the mixed effects models. However, including them in the model had no effect on the results for krill and salps. Therefore, we decided to focus on the two target species *Euphausia superba* and *Salpa thompsoni* in our analysis.

## Results

### Spatial patterns of krill and salp abundance

**Figure 2:**
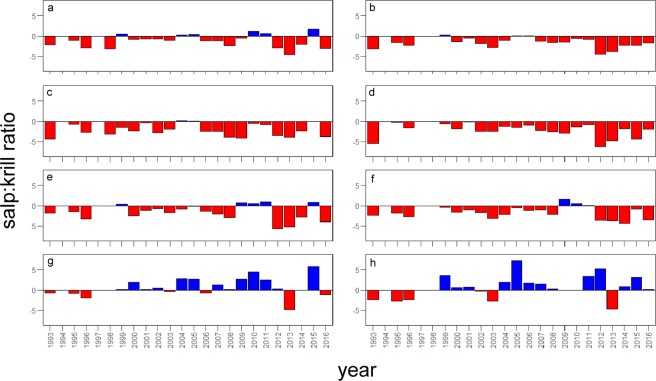
Spatial and temporal patterns of the salp:krill ratio. Negative values (red) indicate higher krill density relative to salps while positive values (blue) indicate more salps in relation to krill. The salp:krill ratio is plotted separately for the North (**a**) and South (**b**) of the Palmer grid as well as the coastal, shelf and slope sub-regions for the northern (**c,e,g**) and southern (**d,f,h**) area, respectively. Note that only data from the reduced dataset (n = 823) were used. In the reduced dataset, concurrent nutrient, krill, and salp abundance data are partially not available for the years 1994, 1997, and 2009.

The salp:krill density ratio revealed significant differences in the regional patterns of co-occurrance and dominance between the investigated areas of the WAP (Fig. 2a). Over the 23 years of observations, krill densities were higher than salp densities across the whole Palmer grid with the exception of a few years with high salp abundance (Fig. 2a).

The partition of the grid into different regions additionally showed a strong variability in the relative densities of krill and salps between north and south as well as coastal, shelf and slope areas. Whereas in the northern area more positive salp:krill ratios were observed over time, indicating higher salp abundance, krill remained more abundant than salps in the southern part of the grid (Fig. 2b). Likewise, krill consistently dominated the coastal and shelf areas (Fig. 2c-f), but salps generally showed significantly higher densities across the slope stations (>1000 m) since 1999 (Fig. 2g,h).

### Relation of krill and salps to dissolved N and P

Across the models, large variation in nutrients (N, P, N:P) was observed between years and when stations were nested within years (Table 1). The random terms accounted for 30–47% of the variance, with most variance being observed between years.

**Table 1:** Results for the random effects from the linear mixed effects models.

Fixed Effect	Random Effects	Response	Variance	Std Dev
Model 1	station:year	N	0.001231	0.03508
	year	N	0.012324	0.11101
	residuals	N	0.01319	0.11485
	station:year	P	0	0
	year	P	0.01203	0.1097
	residuals	P	0.01339	0.1157
	station:year	NP	3.81E-05	0.006176
	year	NP	1.01E-02	0.100322
	residuals	NP	1.19E-02	0.108848
Model 2	station:year	N	0.001221	0.03494
	year	N	0.012443	0.11155
	residuals	N	0.01321	0.11494
	station:year	P	6.02E-12	2.45E-06
	year	P	1.22E-02	1.11E-01
	residuals	P	1.34E-02	1.16E-01
	station:year	NP	1.12E-09	3.35E-05
	year	NP	1.01E-02	1.00E-01
	residuals	NP	1.19E-02	1.09E-01

The table shows the variance and standard deviation of the random effect for each response variable. Model 1 uses log transformed krill and salp abundance as well as the interaction between krill and salps as a fixed effect. Model 2, in turn, used the salp:krill ratio as the fixed effect. In both models, year and station were used as random effects.

N = nitrogen, P = phosphorus, NP = nitrogen:phosphorus ratio.

**Figure 3:**
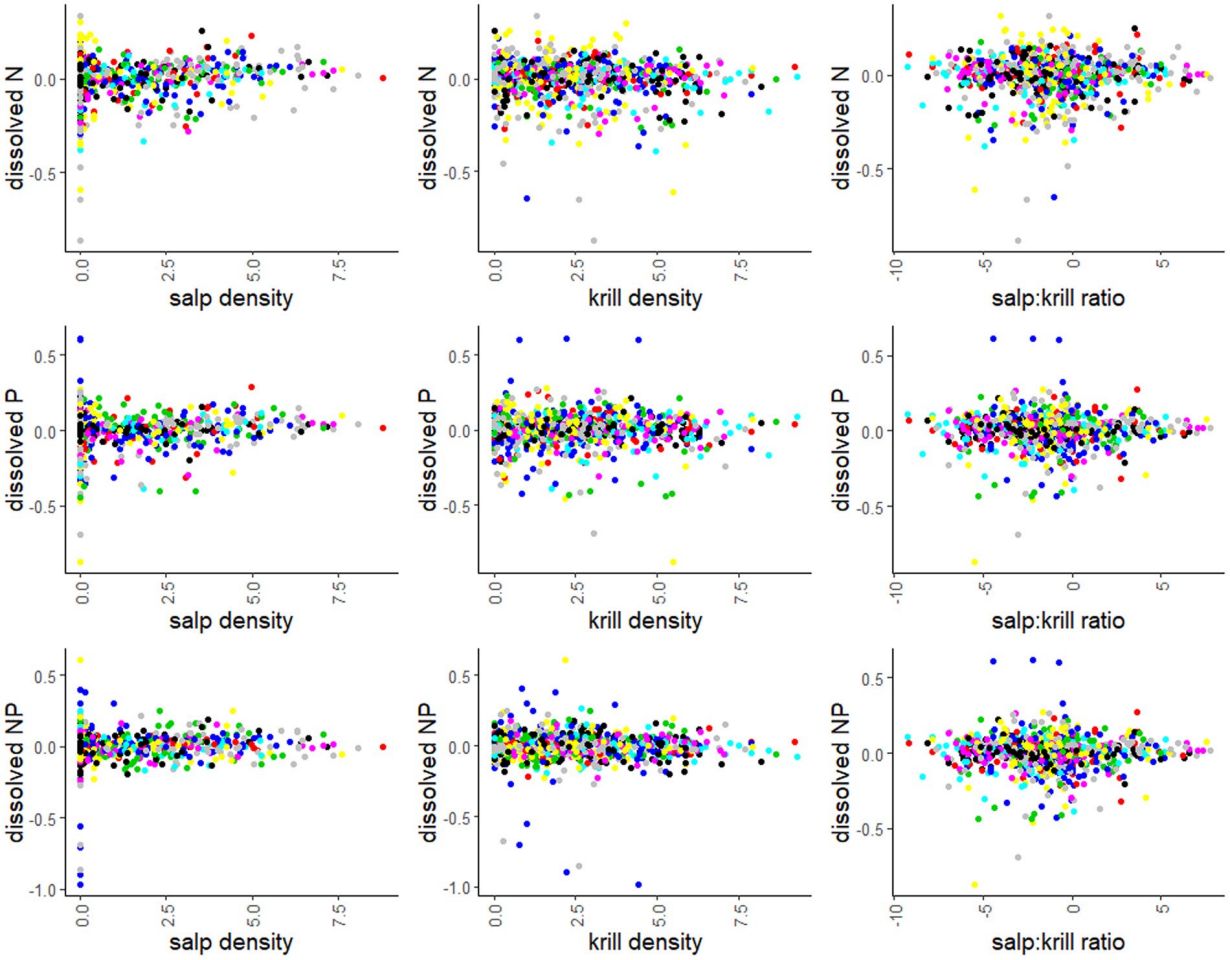
Partial residual plots. Overall relation between dissolved N (µmol), dissolved P (µmol) as well as the N:P molar ratio and the salp and krill density (ind. 1000 m^3^) as well as the salp:krill ratio across the Palmer grid. Note that only data from the reduced dataset (n = 823) were used. The data points display the residuals from the respective linear mixed effects models (model 1 = krill and salp density, model 2 = salp:krill ratio). The colors indicate different years to visualize annual variability. All data are ln-transformed.

However, the mixed effect models revealed significant relationships between krill and salp densities (Model 1) as well as their ratio (Model 2) and the dissolved N and P concentrations as well as the N:P ratios within the Palmer grid between 1993 and 2016 (Table 2). When considering the whole grid, the concentration of dissolved N was significantly lower when more krill were present, while N was positively associated with increasing salp abundance (Table 2, Fig. 3). Likewise, increasing dissolved P concentrations occurred together with high salp densities, but showed no significant relation to krill abundance (Table 2, Fig. 3). Consequently, the dissolved N:P ratio was significantly lower when krill densities increased, while salps tend to be positively associated with the dissolved N:P ratio, although the effect was marginally non-significant (Table 2).

**Table 2:** Results from the linear mixed effect models and the ANOVA.

Fixed Effects	Response	Estimate	Se(Estimate)	T-Value	*F*	p
*Model 1*						
log krill	N	−0.003806	0.002837	−1.341	9.7719	<0.01
log salps	N	0.013654	0.003875	3.524	13.5177	<0.001
log krill*salps	N	−0.002047	0.001564	−1.309	1.7129	0.19
log krill	P	−0.0006484	0.002738	−0.237	2.064	0.15
log salps	P	0.0092009	0.0036838	2.498	6.5431	<0.05
log krill*salps	P	−0.0014792	0.0015146	−0.977	0.9539	0.33
log krill	NP	−0.0042379	0.0025794	−1.643	6.3737	<0.05
log salps	NP	0.0051261	0.0034726	1.476	3.3523	0.06
log krill*salps	NP	−0.0003817	0.0014267	−0.268	0.0685	0.79
*Model 2*						
salp: krill ratio	N	0.007666	0.001629	4.704	22.131	<0.001
salp:krill ratio	P	0.004143	0.001547	2.677	7.1681	<0.01
salp:krill ratio	NP	0.004544	0.001455	3.122	9.7482	<0.01

The p-value was calculated from the *F*-ratio and degrees of freedom. Significant level at p < 0.05. The lmer function automatically calculates t-tests using Satterthwaite approximates to degrees of freedom. For further details on the models see Table 1 or material and methods.

These results became clearer in model 2 through significantly positive relationships between the salp:krill ratio and both dissolved N and P as well as the N:P ratio (Table 2, Fig. 3). A relative increase in salps was correlated with higher concentrations of both dissolved N and P, and – because the effect on N was larger than on P – also led to higher N:P ratios.

We observed regional differences in the direction and magnitude of the relation between krill and salp abundance and N and P between North and South (Appendix Fig. 3 and Fig. 4). When subsetting the dataset into North and South, salp density revealed no significant association with dissolved N, P or the dissolved N:P molar ratio in the northern area of the Palmer grid. Across the southern grid area (south of grid line 400), however, higher salp density was significantly associated with increasing dissolved N, P as well as the N:P ratio.

Contrasting the results for salps, krill density showed a significant negative relation with dissolved N and N:P, but revealed no correlation with P in the northern area. Similar patterns were detected for the South, where high abundances of krill were negatively associated with N, but revealed no significant relation with P or the N:P ratio. The salp:krill ratio, in turn, was positively related to dissolved N and the N:P ratio in the North, i.e. the more salps, the higher the N:P ratio. In the southern part of the Palmer LTER grid the salp:krill revealed a significantly positive relationship with dissolved N and P but not with the N:P ratio. For all fixed effects, i.e. salp density, krill density and the salp:krill ratio, the variance in the data was higher in the South compared to the northern part (Appendix Fig. 3 and Fig. 4). However, when subsetting the whole grid into coastal, shelf and slope areas, we could not detect any significant relations between krill or salps and the dissolved inorganic nutrient pool.

## Discussion

### Spatial patterns of krill and salp abundance

Our results showed that the relative abundance of krill and salps differed most strongly between coastal waters and the slope areas of the Palmer grid. Krill densities were higher than salps along the coast across the grid (north and south), while salps revealed higher densities compared to krill mainly over the slope. The relative abundance data revealed an increasing trend of salp years in the nothern area when the data of the coast, shelf and slope regions were combined. This is, overall, in accordance with previous investigations of the spatial and temporal population dynamics of krill and salps along the west-coast of the Antarctic Pensinsula^3,18,21,23,25^. The consistency of our results with previously documented distribution patterns of krill and salps across the Palmer grid demonstrated that the reduced dataset used in our analysis provided valid information to test our hypothesis regarding dissolved nutrients discussed in the section below.

We deliberately focused our study on krill and salps as they were often described as the dominant macrozooplankton taxa along the WAP, showing high grazing impact and recycling rates. Consequently, our study is mainly aiming at the evaluation of previously stated theoretical assumptions and discussions that shifts in krill and salp populations may influence biogeochemistry at the WAP on a larger scale, and we were mainly interested in whether this can be actually supported by real long-term observational data.

### Impact of krill vs salps on dissolved N and P

The concentrations of nitrogen and phosphorus across the Palmer LTER grid exhibited strong interannual variability and regional differences. Consequently, year and station (used as the random term in our mixed effects model) accounted for roughly 50% of the variability in the nutrient data. Nonetheless, across the whole Palmer grid, the outcome of the models indicated that the concentration of dissolved N and P is significantly positive related with salp densities but negatively correlated with increasing krill densities. Moreover, the more salps were present in absolute or relative terms, the higher the available dissolved N:P ratio. However, we have to mention that the correlation between grazer density and the dissolved nutrients accounted for less than 5% of the overall variability in the N and P data. In addition, the models revealed regional differences in the magnitude and direction of the observed relationships. While the results showed significant differences in the observed relations between the southern and the northern area of the grid, there were no significant relationships along the bathymetrical gradient from the coast to the slope. Although the relative contribution of krill and salp density may be rather small on a larger temporal scale compared to predominant physical drivers, the outcome of the mixed effects model indicates that a dominance shift in the zooplankton compartment from krill to salps can have a significant feedback on the availability and stoichiometry of essential nutrients in the marine ecosystem of the WAP. Our study thereby supports previous assumptions and statements on the future role of krill and salps for the nutrient dynamics along the WAP^22,37,41^. However, the effects of a shift in krill and salp dominance on the dissolved nutrient pool hypothesized in these studies may be less severe than assumed.

The general importance of zooplankton as a biological contributor to the nitrogen and phosphorus required by phytoplankton communities has been recognized in previous field studies and experimental investigations^32,37^. While different abundant zooplankton groups, including microzooplankton, can contribute to the recycling of N and P along the WAP throughout the year^50^, key macrozooplankton grazers such as krill and salps can play a major role in the resupply of N and P to phytoplankton^36,37^. The relation between planktonic grazers and the dissolved nutrient pool in aquatic ecosystems is usually constrained by the stoichiometric demand of the consumer species involved and their species-specific excretion ratios. Several studies from other aquatic systems have pointed out the potentially severe effect of consumer driven changes in nutrient supply on food web dynamics^49,57–59^, and more recent investigations of the WAP marine ecosystem have discussed similar effects^37^. Spatially or temporally heterogeneous aggregations of mobile organisms have the potential to generate biogeochemical hotspots that may modify patterns of nutrient remineralisation and ecosystem nutrient dynamics^60,61^. Thus, dense aggregations of krill and salps may increase nutrient remineralization and therefore concentrations of dissolved N and P on a defined spatial and temporal scale. In a first theoretical approach, Alcaraz *et al*.^41^ estimated the potential consequences of a krill to salp shift for the nutrient dynamics in the SO based on experimental data. They reported higher average N:P excretion ratios in salps compared to adult krill and concluded that the average metabolic N:P ratio of the whole zooplankton community will increase by a factor of two in case of a persistent krill to salp shift. In fact, the available data on krill and salp stoichiometry indicate lower N:P excretion ratios compared to body elemental composition in both species^34,45^, indicating that N is retained preferentially in krill and salps. On average, however, the N:P excretion ratio of salps can be higher compared to krill^41^. This may indicate relatively higher P demand in salps due to higher growth rates and less N-rich structural compounds. Other studies on invasive species have shown that an increase in the population of organisms with specific stoichiometric footprints that differ from the native community significantly altered the nutrient dynamics by changes in remineralization rates. A study from the Caspian Sea, for instance, demonstrated increased nitrogen concentrations after the intrusion of the ctenophore *Mnemiopsis leidyi*^62^. This shows that changes in consumer composition can significantly modify N and P dynamics in aquatic ecosystems with subsequent consequences for element fluxes and biogeochemical cycles.

One might argue, that inorganic N and P are usually available in excess around the WAP and consequently may not be the main limiting nutrients for primary production^4^. However, along the coast of the WAP, micronutrient concentrations are often high enough to promote phytoplankton blooms that may completely utilize the available macronutrients^63–65^, making phytoplankton communities more vulnerable to changes in recycled dissolved N:P ratios. Even under nutrient replete conditions, changes in the optimal N:P ratio available for the phytoplankton community can modify the composition and productivity of primary producers^66^. In addition to that, Glibert *et al*.^57^ highlighted the importance of macronutrients such as N and P not only at levels of limitation but also at excess concentrations relative to the cellular demand. Consequently, eleveated N:P ratios of the inorganic nutrient pool due to altered zooplankton community excretion ratios can exert a strong feedback effect on phytoplankton community structure, indicating that a shift from krill to salps as the dominant macrozooplankton grazers may select for phytoplankton communities that exhibite higher N:P ratios. Studies on the relation between POM stoichiometry and phytoplankton community structure in the SO reported lowest seston N:P ratios in areas dominated by diatoms and, in contrast, relatively high N:P where flagellates or *Phaeocystis* dominated^67–69^. Thus, potential modifications of the inorganic N:P pool are likely to further accelerate the reported changes in phytoplankton community composition along the WAP^10,11^. In addition, nutrient dynamics in the SO are often closely coupled to bloom variability and the nutrient drawdown by phytoplankton^63,67,70^. Consequently, changes in inorganic N:P and subsequent shifts in phytoplankton community structure may substantially alter the draw down and export ratios of N:P to deeper layers and further modify the biogeochemistry and food web dynamics of the WAP^67,70^.

Differences in the species specific grazing efficiency of krill and salps represents another potential mechanism that may explain changes in nutrient dynamics. It has been described that krill predominantly consumes diatoms while salps, in turn, show a higher efficiency for smaller food particles such as flagellates^39^. Such differences in prey-specific consumption of stoichiometrically different phytoplankton taxa may influence the nutrient draw down by phytoplankton and subsequently change the stoichiometry of phytoplankton communities and dissolved nutrients. Aside from nutrient changes induced by trophic interactions in the plankton community, physical factors such as vertical mixing also play an important role for nutrient dynamics, especially on the slope and the continental shelf of the WAP. Upwelling and mixing of the Circumpolar Deep Water (CDW) pumps nutrient rich water onto the continental shelf, potentially reducing the relative importance of macrograzer recycling for the long-term dynamics of dissolved nutrients^5,64^.

However, potential changes in the N:P supply may become more relevant in future scenarios for coastal areas of the WAP, where shelf sediments and glacial run-offs may increase trace metal inputs, sufficient enough to promote substantial phytoplankton growth^71,72^. In addition, upwelling and nutrient fluxes may be reduced with increasing temperatures and a stabilizing thermocline, thereby potentially creating nutrient limitation and higher vulnerability of phytoplankton to shifts in dissolved N:P ratios within the WAP marine ecosystem. Considering continuously increasing salp densities along the WAP, the coastal ecosystem may display elevated dissolved N:P ratios in the future with subsequent consequences for nutrient dynamics and phytoplankton community composition.

## Conclusion

Based on long-term observational data, our findings indicate for the first time that changes in dense populations of major macrograzers such as Antarctic krill and salps can be related to patterns in N and P stoichiometry along the western coast of the Antarctic Peninsula. Although resource availability at the WAP shows high temporal and spatial variability that is predominantly driven by seasonality, geomorphology and the unique hydrographic patterns, the outcome of our analysis provides first evidence from existing long-term observational data, that changes in the relative abundance of krill and salps have the potential to alter the N:P dynamics along the coast of the WAP. Thereby, our results support previous assumptions on the relation between predicted changes in krill and salp densities and the biogeochemistry of the Southern Ocean.

However, at the current state, information on stoichiometric dynamics in planktonic systems of remote areas like the WAP remain limited and therefore conclusions are still speculative. We therefore hope, that our study provokes further discussion and investigations of the stoichiometric interactions in planktonic communities of the SO. We further emphasize the importance of a comprehensive assessment of plankton community structure and biogeochemistry in order to predict how future changes in the composition of key species might modify stoichiometric dynamics in the planktonic food web of the Southern Ocean.

## Supplementary information

Supplementary Information.

## Data Availability

The datasets analysed during the current study are available on the Palmer LTER webpage https://pal.lternet.edu/data. The data and analysis generated during the current study are available from the corresponding author on reasonable request.
