# Supplementary material for: Krill vs salps: dominance shift from krill to salps is associated with higher dissolved N:P ratios
Source: Sci Rep. 2020 Apr 3;10:5911. doi: 10.1038/s41598-020-62829-8 (PMC7125175; doi:10.1038/s41598-020-62829-8)
Supplement: Supplementary file 1 — Supplementary Information. [file 41598_2020_62829_MOESM1_ESM.pdf]

Krill vs salps: dominance shift from krill to salps is associated with higher dissolved N:P ratios

Christoph Plum[1*], Helmut Hillebrand[1,2,3,] and Stefanie Moorthi[1]

[1]University of Oldenburg, Institute for Chemistry and Biology of the Marine Environment (ICBM), Wilhelmshaven, Germany

2 Helmholtz Institute for Functional Marine Biodiversity (HIFMB) at the University of Oldenburg, Oldenburg, Germany

3 Alfred Wegener Institute, Helmholtz-Centre for Polar and Marine Research (AWI), Bremerhaven, Germany

[*]Corresponding Author: c.plum@uni-oldenburg.de

Supplementary Information

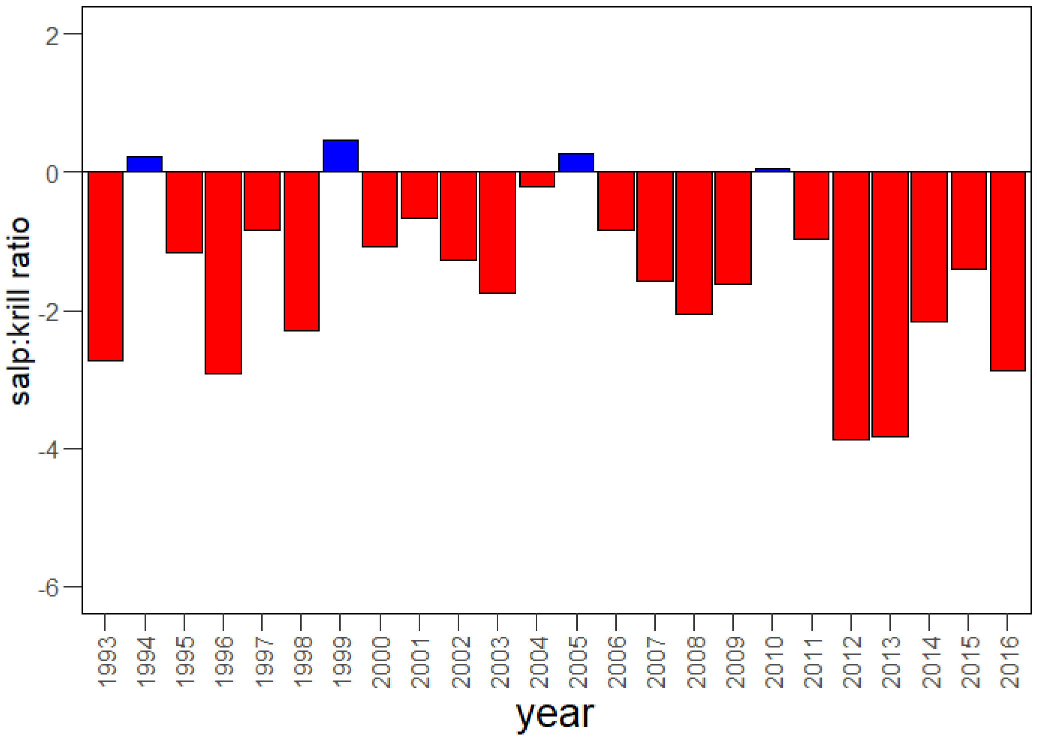

Appendix Figure 1: Temporal patterns of the salp:krill ratio between 1993 and 2016. Data include all krill and salp density values from the whole zooplankton dataset (n = 1243). Negative values (red) indicate higher krill density relative to salps while positive values (blue) indicate more salps in relation to krill.

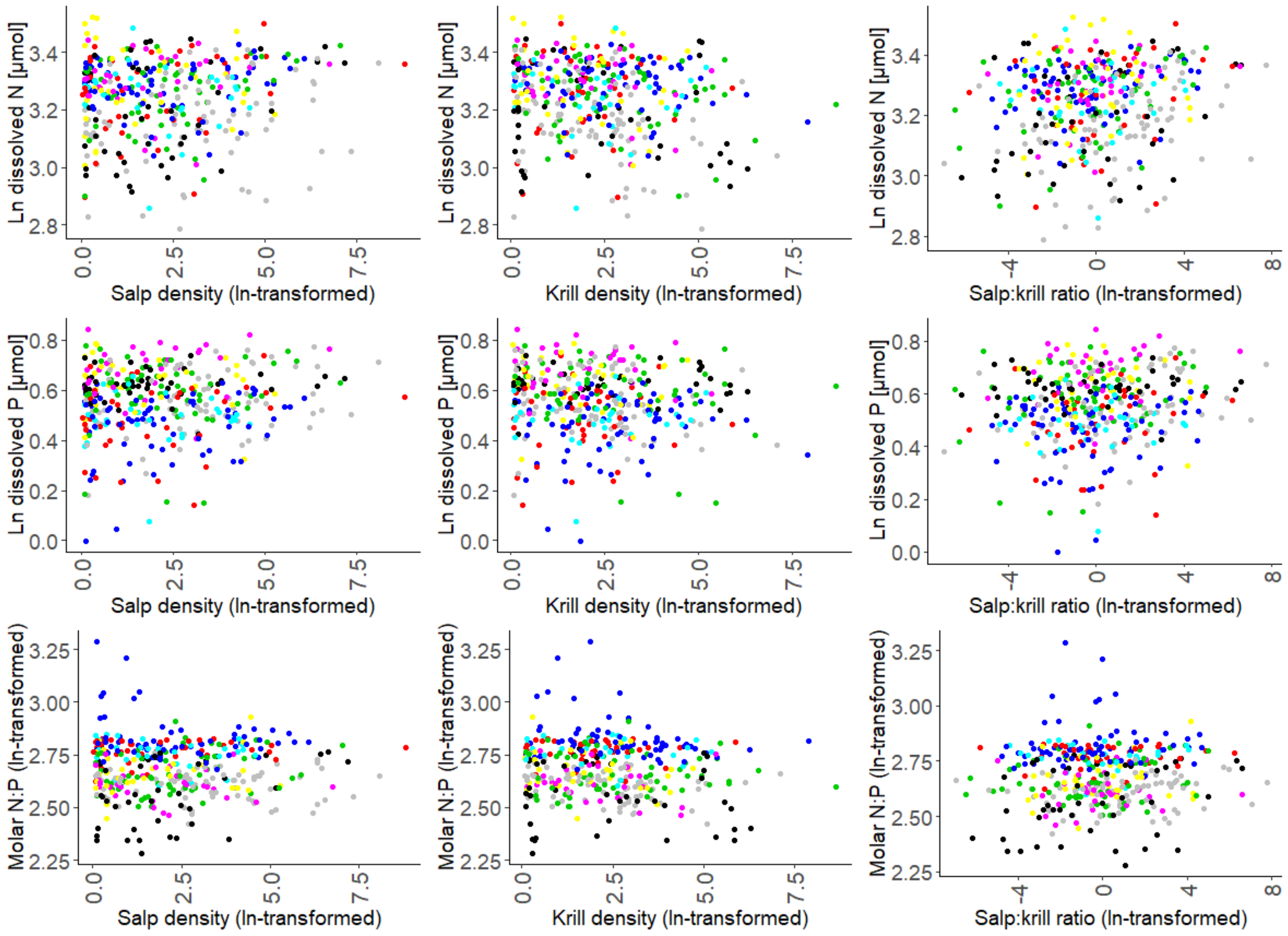

Appendix Figure 2: Overall relation between dissolved N, dissolved P and the N:P molar ratio and the salp and krill abundance as well as the salp:krill ratio across the Palmer grid. Note that only data from the reduced dataset (n=823) were used.

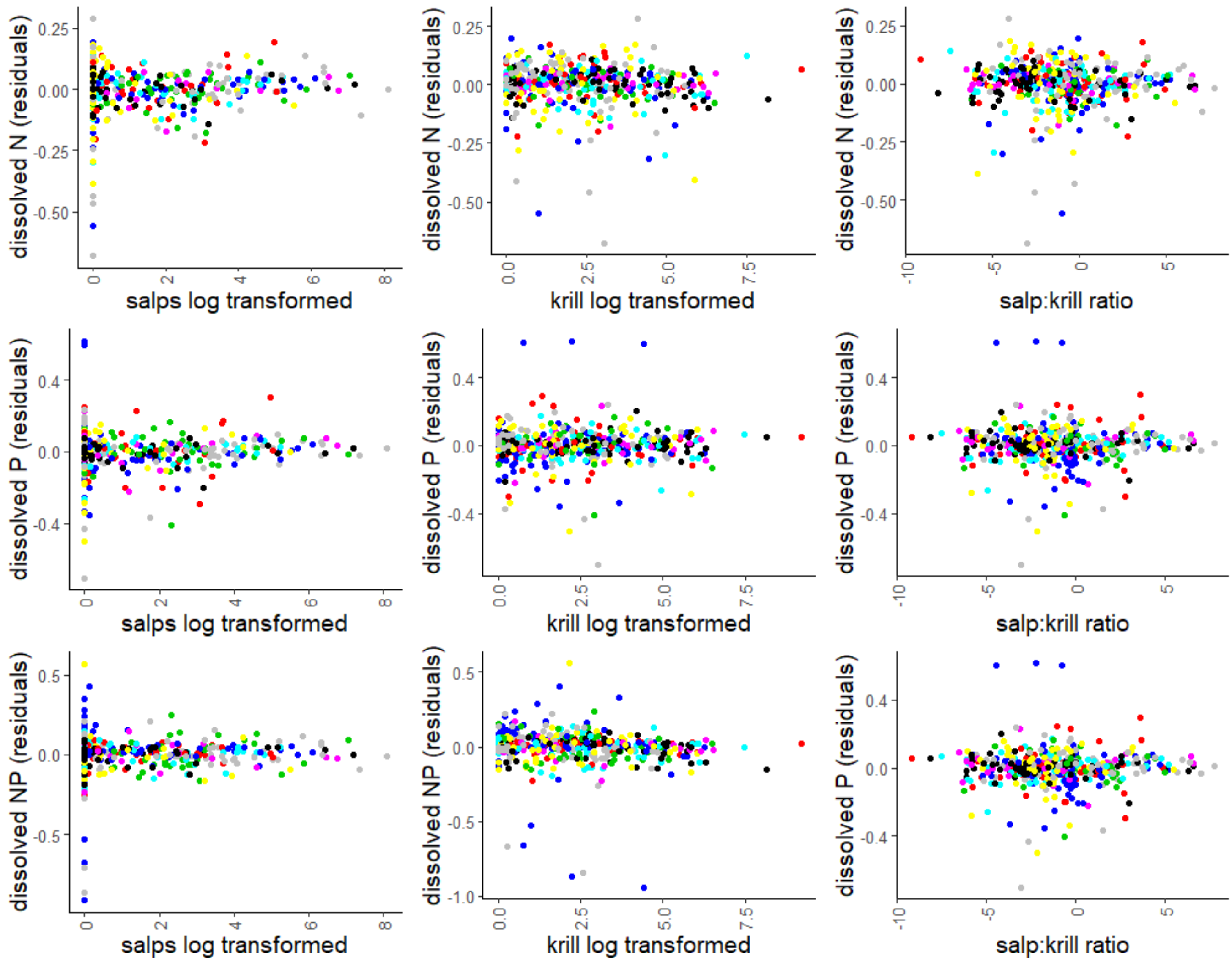

Appendix Figure 3: Partial residual plots of the northern area. Relation between dissolved N, dissolved P and the N:P molar ratio and the salp and krill density (ind. $1000m^3$) as well as the salp:krill ratio in the northern part of the Palmer grid. Note that only data north of grid line 400 of the reduced dataset (n=823) were used. The data points display the residuals from the respective mixed effect models.

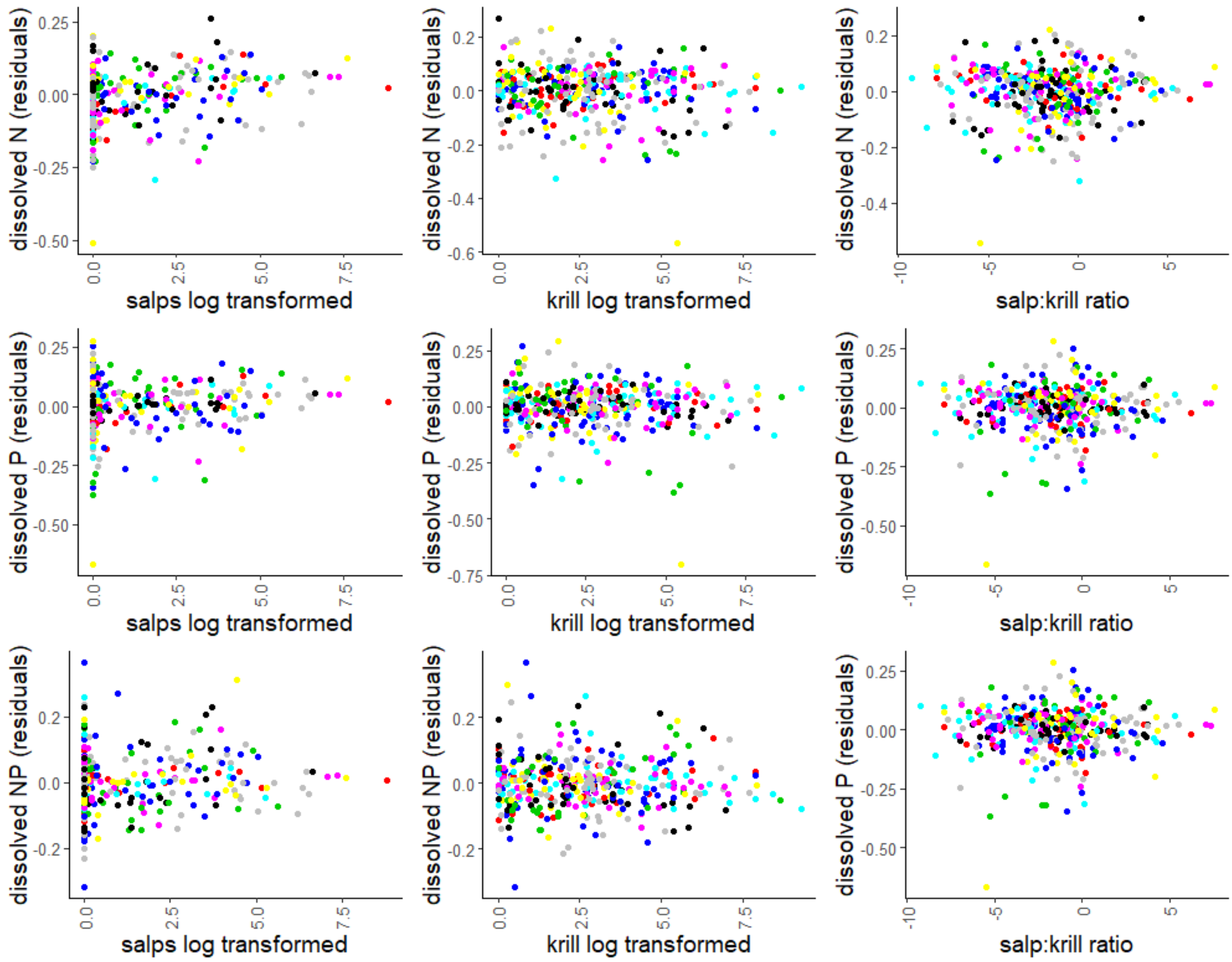

Appendix Figure 4: Partial residual plots of the southern area. Relation between dissolved N, dissolved P and the N:P molar ratio and the salp and krill density (ind. 1000m^3) as well as the salp:krill ratio in the southern part of the Palmer grid. Note that only data south of grid line 400 of the reduced dataset (n=823) were used. The data points display the residuals from the respective mixed effect models.
